# A novel GAN-based regression model for predicting frying oil deterioration

**DOI:** 10.1038/s41598-022-13762-5

**Published:** 2022-06-21

**Authors:** Kai Ye, Zhenyu Wang, Pengyuan Chen, Yangheran Piao, Kuan Zhang, Shu Wang, Xiaoming Jiang, Xiaohui Cui

**Affiliations:** 1 Key Laboratory of Aerospace Information Security and Trusted Computing, Ministry of Education, School of Cyber Science and Engineering, Wuhan University, Wuhan, China; 2 Wuhan Institute for Food and Cosmetic control, Wuhan, China; 3 Jiaxing Future Food Research Institute, Jiaxing, China

**Keywords:** Public health, Computer science

## Abstract

Frying is a common food processing method because fried food is popular with consumers for its attractive colour and crisp taste. What’s concerning is that the complex physical and chemical reactions occurring during deep frying are harmful to the well-being of people. For this reason, researchers proposed various detecting methods to assess frying oil deterioration. Some studies design sensor probe, others utilize spectroscopic related methods. However, these methods all need the participating of professionals and expensive instruments. Some of the methods can only function on a fixed temperature. To fix the defects of the above models, in this study, we make use of recent advances in machine learning, specifically generative adversarial networks (GAN). We propose a GAN-based regression model to predict frying oil deterioration. First, we conduct deep frying experiments and record the values of indexes we choose under different temperature and frying time. After collecting the data, we build a GAN-based regression model and train it on the dataset. Finally, we test our model on the test set and analyze the experimental results. Our results suggest that the proposed model can predict frying oil deterioration without experiments. Our model can be applied to other regression problems in various research areas, including price forecasting, trend analysis and so on.

## Introduction

Frying is a commonly used food processing method because fried food are popular with consumers for their attractive color and crisp taste. However, during deep frying, complex reactions will occur which leads to the physical and chemical properties change of the frying oil. As a result, the quality and safety of the frying oil might change^1,2^. Therefore, the research concerning the quality and safety of frying oil has attracted the attention of many researchers^3^.

During high temperature frying, various chemical reactions such as oxidation, hydrolysis and polymerization occur continuously^4,5^. After a set of chain reactions^6^, short compounds including a series of small molecular alcohols, aldehydes, ketones, acids and lactones are generated^7^. With the unceasing occurrence of above chemical reactions, some harmful substances are generated and accumulated, leading to the deterioration of frying oil^8^. Consequently, some indexes reflecting the physical and chemical changes of frying oil can be used for detection of oil deterioration.

Total polar compound (TPC) composed of multiple substances is a widely used evaluation index for frying oil quality^9^. Many countries have regulations on the content of TPC in frying oil. Germany limits the content of TPC to no more than 24%, some European countries (France, Italy ...) are 25%, China and Switzerland are 27%^10^. Thus, we use TPC in this paper as an evaluation metrics. Besides TPC, some other evaluation metrics are also commonly used. Acid value (AV) is another popular metrics for evaluation of oil degradation during frying^11,12^. Among those evaluation metrics, we choose TPC, AV, trans fatty acids (TFA)^13–15^ and triacylglycerol polymers (TGP)^6,16^. In order to determine these metrics, researchers have proposed various methods. Column chromatography^17^ is frequently used to detect TPC content in frying oil. While being accurate, the detection process is time-consuming and has to be done by experts^18^. In recent years, Near infrared spectrum (NIRS) technology^19,20^ has been accepted on food quality analysis for its fast and non-destructive detection. For instance, Cascant et al.^21^ and Kuligowski et al.^22^ proposed alternative methods for detecting TPC and TGP in frying oil based on NIRS and PLSR. Though NIRS technology has been proven effective in edible oil detection^23^, it still faces some challenges. With the extension of frying time, the composition of frying oil changes complexly. As a result, near-infrared spectrum is difficult to accurately reflect the changes of its comprehensive quality with one or several parameters or indicators. Also, NIRS technology still needs instruments and professionals to conduct experiments. It is hard for non-professionals to get access to those resources.

This study aims to construct a model to predict the deterioration of frying oil without expensive instruments and experiments. Previous study^24^ has successfully established a model to forecast carbonyl value of frying oil using traditional regression methods. However, due to the limit of the regression methods they used, their model can only function at a fixed temperature. This is apparently a major disadvantage.

Recently, the development of neural networks^25^ has attracted the attention of many researchers from various research fields. Among these advances, generative adversarial networks (GAN)^26^ has shown its great ability at generating adversarial images in computer vision. Inspired by its idea, we explore modifying the original GAN for regression and apply it to the prediction of frying oil deterioration.

In this study, we propose a GAN-based regression model to predict frying oil deterioration. First, we conduct deep frying experiments and record the values of indexes we choose under different temperature and frying time. After collecting the data, we build a GAN-based regression model and train it on the dataset. Finally, we test our model on the test set and analyze the experimental results. Our results suggest that the proposed model can predict frying oil deterioration without experiments.

Besides being applied to food safety, our model can be applied to other regression task as well, including financial problems as price forecasting, trend estimation and managerial applications such as data analysis of human resource management.

Our contributions can be summarised as follows:

- We incorporate generative adversarial network to improve the general performance of regression model in the task of frying oil deterioration. As far as we are concerned, we are the first to do so.
- We propose a novel method to automatically predict whether the frying oil has degraded without the participating of professionals and our proposed method surpasses several classical methods in terms of popular evaluation metrics.

## Materials and methods

### Deep frying experiment

#### Materials and instruments

Palm oil, rice, soybeans, flour and salt are all purchased in the market; Anhydrous ethanol, 95% ethanol, isopropanol, ether, methyl tert butyl ether, phenolphthalein, petroleum ether, acetone, potassium hydroxide, sodium bisulfate, sodium chloride, anhydrous sodium sulfate, etc. (all analytical pure); Methanol, isooctane, tetrahydrofuran, acetonitrile, n-hexane, n-heptane, ethyl acetate and dichloromethane (all chromatographic pure).

The equipment used in the deep frying experiment includes: Eopc automatic edible oil polar component separation system and flash chromatographic column (Tianjin bonaijer Technology Co., Ltd), Waters 2695 high performance liquid chromatograph and 2414 differential refractive detector (Waters), Agilent 7890 gas chromatograph (Agilent, USA), Centrifuge 5810R freezing centrifuge (Eppendorf, Germany), Ultrasonic cleaner (Prima, UK) and frying equipment (Demashi L-102C).

#### Frying process

After washing the rice and soybeans with water, they were soaked for 12 h. Then we took out the soaked rice and soybeans respectively, rinsed them with clean water. After that, we poured the rice, soybeans and water into the wall breaker at the ratio of 3:1:2. Next, we stirred the mixture and sifted it, filtered out the bean residue and other impurities. We added an appropriate amount of flour into it and stirred them into mush. The mush is then sifted. Finally, we added an appropriate amount of salt and scallion, stirred them evenly, covered with plastic wrap and let it rise for 30 min to 1 h for frying.

During frying, we first added about 5L palm oil into the frying equipment, heated and raised the temperature to the set temperature. Then we added 80g ± 5g material obtained in the above process and put it into the equipment for frying. When the fried mush became solid and the two sides of fried mush turned golden, we removed it, and the obtained mass is 50 g ± 5 g. At the same time, we put in new frying material and continued frying. We repeated this process to keep the frying for 9 h every day and conducted continuous 4-day intermittent atmospheric pressure frying for 36 h in total.

For the experiment, we didn’t add new oil during frying. Raw oil samples were taken before frying. During frying, 50ml oil samples were taken every 4 h. We got several frying oil samples at each set temperature, and stored them at − 20 °C temperature to determine the acid value, polar components, triglyceride polymer, fatty acid, polycyclic aromatic hydrocarbons, chloropropyl alcohol ester and other quality indexes of the obtained frying oil samples, for analyzing the correlations and changes of frying oil quality.

It is worth mentioning that during deep frying experiment, we also recorded other indexes like PAH4 and Benzopyrene. However, these indexes either change irregularly or do not change significantly. Thus, we only use the four indicators mentioned above (TPC, AV, TFA and TGP) for prediction.

#### Methods of measurement

AV is determined according to method one of GB 5009.229-2016; TPC is determined according to method one of GB 5009.202-2016; TGP was determined according to DB 34 / T 1997-2013; TFA is determined according to method three of GB 5009.168-2016.

### GAN-based regression model

**Figure 1:**
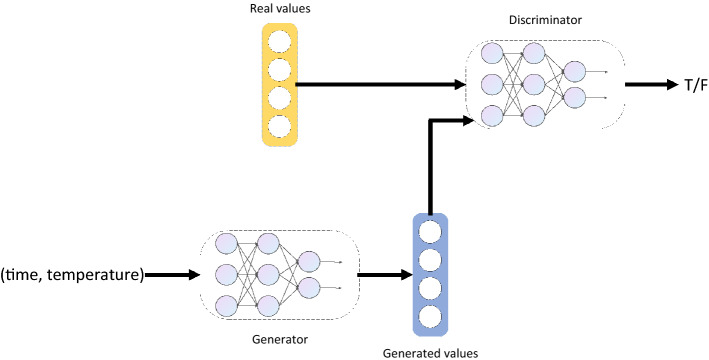
The framework of our proposed model.

The objective of this paper is to establish a model for frying oil deterioration without professionals or expensive devices. Therefore, it is vital to construct a regression model to predict values of selected indexes given frying time and temperature. Here, we discuss our GAN-based regression model in detail. As shown in Fig. 1, our model consists of two modules, Generator and Discriminator. In Generator module, we apply a feed-forward neural network (FNN) to process the input of frying time and temperature. The Generator module will generate the predicted values of indexes. After that, either the predicted values or real values recorded in the deep frying experiments will be fed into the Discriminator module. The Discriminator module, also an FNN, will try to discern whether the values are real or not and will finally output a probability value denoting it. When we complete the training, given frying time and temperature, we use the Generator module to predict the values of indexes and decide whether the frying oil will deteriorate on that condition.

#### Training procedure

We apply adversarial training to train the GAN-based model. The procedure is formally presented in Algorithm 1. When the training completes, we use the Generator for regression.
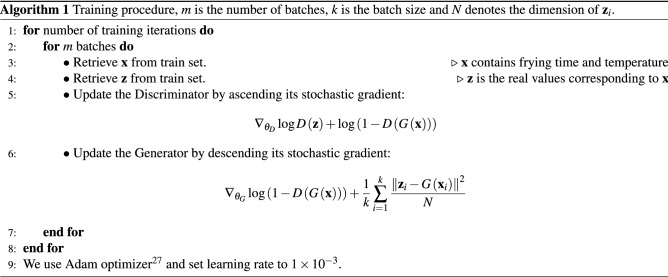


### Determination of oil deterioration

After establishing our regression model, we can acquire the predicted values of indexes on given frying time and temperature. If one of the values surpasses the threshold we set, we determine that the oil will deteriorate on that condition. The thresholds are set to:

$$\begin{aligned} ( AV _{threshold}, TPC _{threshold}, TGP _{threshold}, TFA _{threshold}) = (5, 27, 10, 2) \end{aligned}$$

## Results

### The proposed framework

Figure 1 shows the framework of our proposed model. Similar to traditional GAN, our framework consists of two modules, Generator and Discriminator. In Generator module, we apply a feed-forward neural network (FNN) to process the input of frying time and temperature. The Generator module will generate the predicted values of indexes. After that, either the predicted values or real values recorded in the deep frying experiments will be fed into the Discriminator module. The Discriminator module, also an FNN, will try to discern whether the values are real or not and will finally output a probability value denoting it. When we complete the training, given frying time and temperature, we use the Generator module to predict the values of indexes and decide whether the frying oil will deteriorate on that condition. In “Materials and methods” section, we describe the details of our model and the training procedure.

### Dataset

**Figure 2:**
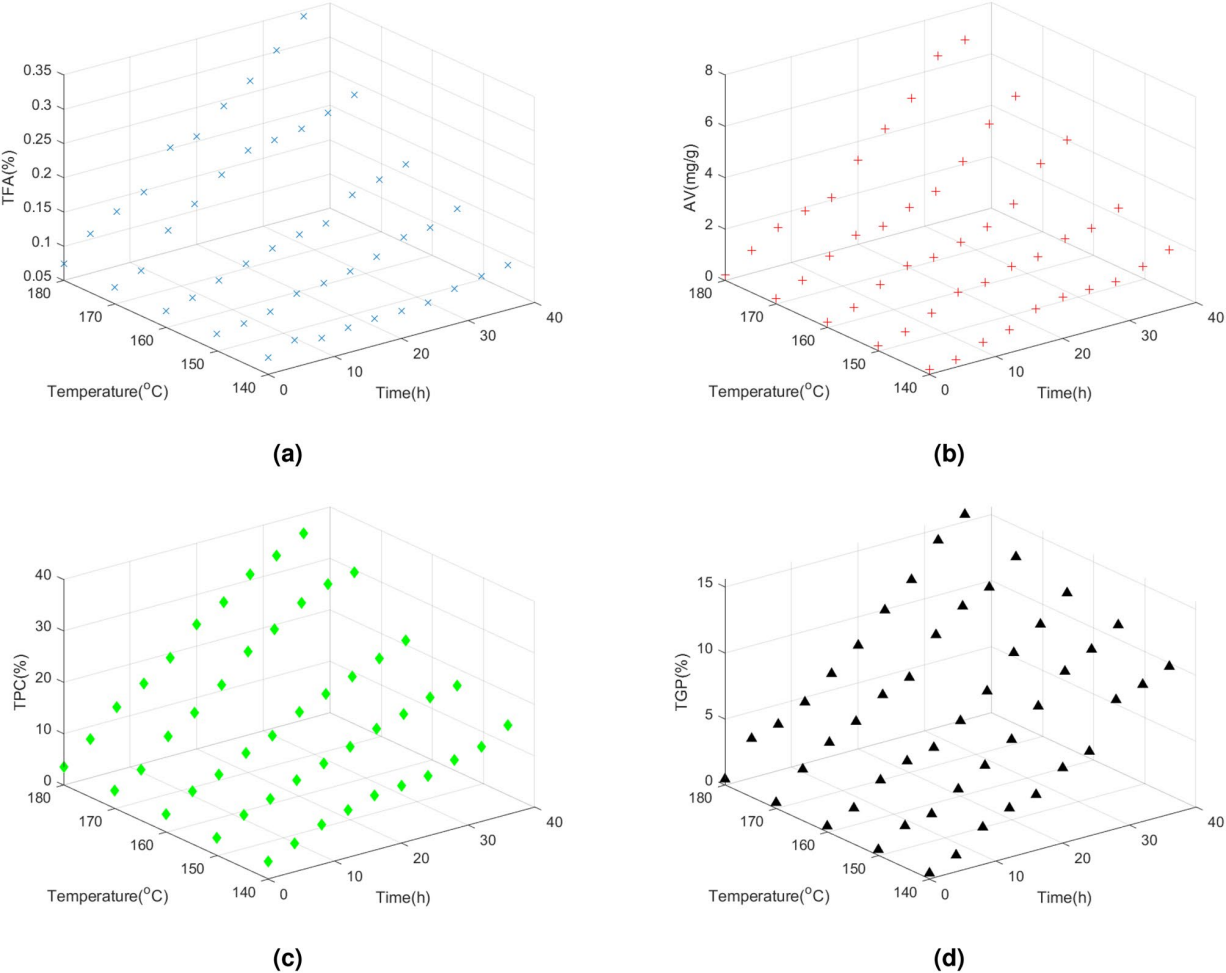
The above four figures show the correlation between frying time and temperature with the four indexes.

We conduct deep frying experiments and record the value of chosen indexes on different frying time and temperature. We record the values of TPC, AV, TFA and TGP. Frying temperature range from 140 to 180 °C. The maximum of frying time is set to 36 h. In total, we record 200 values. The specific details of our dataset is shown in Fig. 2. As is presented in the figure, with the increase of frying time and temperature, the four indexes increase as well. When one of them reach the threshold, we can decide the oil has deteriorated.

### Results of prediction

**Figure 3:**
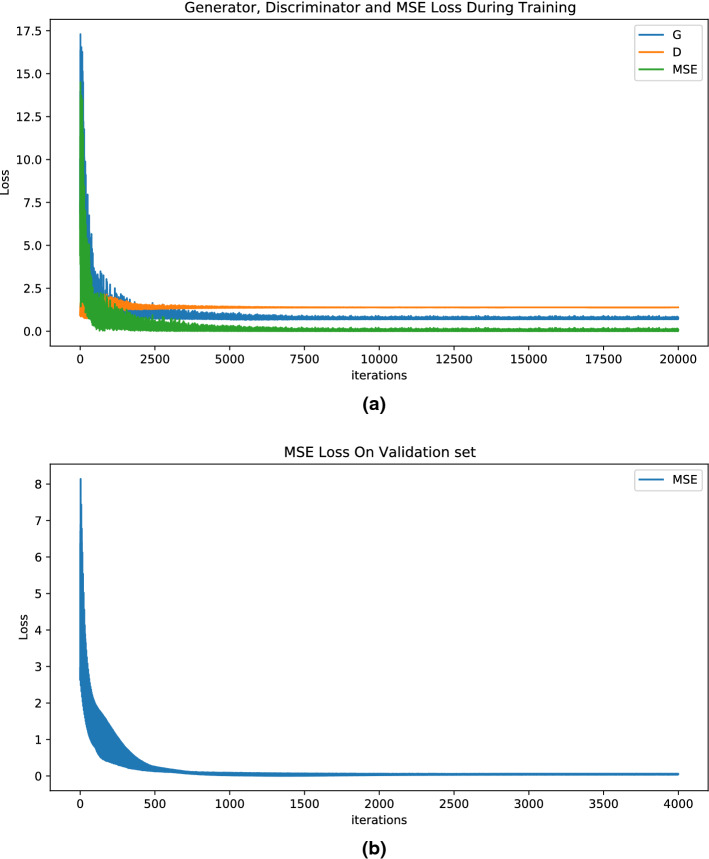
The above figures show the losses on train set and validation set specifically.

Figure 3 demonstrates the losses on train set and validation set specifically. In Fig. 3a, blue, yellow and green points denote loss of Generator, loss of Discriminator and MSE loss respectively. While in Fig. 3b, as we use Generator for regression, we utilize MSE loss for evaluation. Thus the blue points in Fig. 3b denote MSE loss on validation set. The details of loss functions are explained in “Materials and methods” section. As shown in Fig. 3, with the iterations increasing, the losses on both sets first decline drastically then keep steady.

When the training completes, we evaluate the performance of our model on test set. The real values versus the predicted values of chosen indicators are shown in Table 1. We can easily observe from the table that, with the increase of frying time and temperature, the four indicators increase as well. The predicted values also conforms to this trend, demonstrating that our model is effective at learning the hidden features.

It is worth mentioning that in Fig. 3, we set the *x*-axis to the number of iterations instead of epochs to better visualize the results.

**Table 1:** The real values versus the predicted values of chosen indicators.

Time(h)	Temperature (°C)	Real values				Predicted values			
		AV (mg/g)	TPC (%)	TGP (%)	TFA (%)	AV (mg/g)	TPC (%)	TGP (%)	TFA (%)
4	150	0.4653	6.5000	1.7100	0.0796	0.0019	6.6604	1.0424	0.2914
8	180	1.4942	12.4000	3.5000	0.1292	2.0568	16.2901	4.2830	0.1701
8	140	0.7041	7.8000	2.8400	0.0811	− 0.0706	6.2470	1.8503	0.2956
16	140	1.4387	10.7000	4.2100	0.0892	0.8481	10.4405	4.6824	0.2419
20	170	2.3514	23.6000	7.1900	0.2210	2.9030	20.0702	7.9230	0.1206
28	140	1.6464	13.4000	9.7500	0.1009	2.2261	16.7313	8.9303	0.1611
28	150	2.3987	17.7000	10.1200	0.1412	2.7580	19.2421	9.5388	0.1297

If one of the values surpasses the threshold, we determine that the oil will deteriorate on that condition. The thresholds of indicators are set to: $$( AV _{threshold}, TPC _{threshold},$$ $$TGP _{threshold}, TFA _{threshold}) = (5, 27, 10, 2)$$.

### Statistical Evaluation

In order to compare the performance of our proposed model and existing methods, we carefully select two classic algorithms for time series regression: the moving average (MA)^28^ and vector autoregression (VAR)^29^. Also, we choose some popular evaluation metrics to measure the performance of the models: Mean Absolute Scaled Error^30^(MASE), Mean Squared Error^31^(MSE) and Mean Absolute Error^30^(MAE).

**MAE**: The mean absolute error is a measure of errors between predicted values and real values. It is calculated as the following:

$$\begin{aligned} {\text {MAE}}=\frac{1}{n} \sum _{i=1}^{n}\left| y_{i}-{\hat{y}}_{i}\right| \end{aligned}$$

where ${\hat{y}}_{i}$ is the predicted value and $y_{i}$ is the corresponding real value.

**MSE**: the mean squared error measures the average squared difference between the predicted values and the real value. It is calculated as the following:

$$\begin{aligned} \mathrm {MSE}=\frac{1}{n} \sum _{i=1}^{n}\left( y_{i}-{\hat{y}}_{i}\right) ^{2} \end{aligned}$$

where ${\hat{y}}_{i}$ is the predicted value and $y_{i}$ is the corresponding real value.

**MASE**: The mean absolute scaled error is a measure of the accuracy of forecasts. It is the mean absolute error of the forecast values, divided by the mean absolute error of the one-step naive forecast. It is calculated as the following:

$$\begin{aligned} {\text {MASE}}=\frac{1}{n} \frac{\sum _{j=1}^{n}\left| y_{j}-{\hat{y}}_{j}\right| }{\frac{1}{n-1} \sum _{i=2}^{n}\left| y_{i}-y_{i-1}\right| } \end{aligned}$$

where ${\hat{y}}_{i}$ is the predicted value and $y_{i}$ is the corresponding real value.

The result of statistical evaluations is shown in Table 2. As is shown the Table 2, in terms of all the evaluation metrics, our proposed model surpasses the competing models, demonstrating the effectiveness of our method. It is worth mentioning that during statistical evaluation, the split of datasets is a little different. We select indicators of 26 h and 28 h as test set because the competing models are time series models, and randomly selecting test set will negatively influence their performances.

**Table 2:** The above table shows the result of statistical evaluations.

Model Name	MASE (Mean Absolute Scaled Error)	MSE (Mean Squared Error)	MAE (Mean Absolute Error)
MA	3.852	33.096	4.051
VAR	1.776	10.929	1.671
GAN-R (our proposed)	1.664	4.084	1.256

## Discussion and conclusion

In this study, we propose a GAN-based regression model to predict frying oil deterioration without the participating of professionals and expensive instruments. While frying is a popular food processing method globally, the complex reactions during frying lead to the deterioration of frying oil^6^, threatening the health of customers. For this reason, researchers proposed indicators and detecting methods to assess oil deterioration. Some studies designed sensor probe for frying oil deterioration^32^. In their study, the researchers designed a capacitive sensor using interdigitated electrode structure to measure changes on its electrical capacitance during frying. Other study utilizes a rapid near-infrared (NIR) spectroscopic method to measure products of chemical reactions in frying oil, including total polar materials (TPMs) and free fatty acids (FFAs)^19^. And then they use those measured indicators to determine frying oil degradation. These methods above all need the participating of professionals and expensive instruments. Recent studies^17,24^ construct regression models to automatically predict carbonyl value of frying oil given time value. Although the models of Liu et al.^17,24^ show great performance, their model can only function at a fixed temperature due to the limit of regression methods they used. This study follows this line of research with further improvement.

To fix the defects of the above models, in this study, we make use of recent advances in machine learning, specifically generative adversarial networks (GAN)^26^. Though GAN was originally proposed as a kind of generative model for image generation, different variants of GANs later came out for other tasks^33–38^. Following previous research line, we modify original GAN structure for regression. Details of our proposed model will be explained in “Materials and methods” section. We assume our GAN regression model surpasses previous methods in mainly two aspects; on the one hand, our model doesn’t need professionals and instruments like the previous probe-based method or has the limitation of functioning at a fixed temperature. On the other hand, incorporating GAN as the regression model can improve accuracy and enhance generalization ability. Therefore, as is described in “Results of prediction” section, we conducted experiments to demonstrate our assumptions. Here, we give a thorough analysis of the experimental results.

According to Fig. 3a, after five thousand iterations, the training process is almost completed. In the meantime, the steadiness of Generator loss and Discriminator loss is well predicted because of the inherent characteristics of GANs. MSE loss is also close to zero as iterations increase. The steadiness of MSE loss on validation set (in Fig. 3b) along with what can be seen in Fig. 3a denotes the success of the training process. Then we evaluate the effectiveness of our model on test set. From Table 1, we can observe that the disparity between real values and predicted value is rather small when the values of indicators increase. And for frying oil deterioration, our model basically predicts the right result. However, it can’t be neglected that the prediction accuracy is not very desirable especially when the value of indicators becomes close to zero. This problem might be addressed in future research. One possible solution is to modify the network architecture and hyperparameters.

## Data Availability

The dataset used during the current study is available without any restrictions from the corresponding author on reasonable request.
